# Global Perspectives on Physician-Assisted Death: A Cross-Sectional Survey of Doctors’ Opinions

**DOI:** 10.7759/cureus.97464

**Published:** 2025-11-21

**Authors:** Cassandra Clara Ramadass, Mukund Suresh, Vedamurthy Adhiyaman, Shanti Gautam, Shantanan Bathumalai, Kisshan Raj Balakrishnan

**Affiliations:** 1 Care of the Elderly, Glan Clwyd Hospital, Bodelwyddan, GBR; 2 Emergency Medicine, East Lancashire Hospitals NHS Trust, Blackburn, GBR; 3 Internal Medicine, Glan Clwyd Hospital, Bodelwyddan, GBR; 4 Pediatrics, Wrexham Maelor Hospital, Wrexham, GBR; 5 Urology, Wrexham Maelor Hospital, Wrexham, GBR

**Keywords:** assisted dying, autonomy, cross-sectional survey, euthanasia, global, medical assistance in dying, medical ethics, physician assisted death, physician assisted suicide, voluntary assisted dying

## Abstract

Introduction

Physician-assisted death (PAD) remains ethically and legally contested worldwide. PAD encompasses both euthanasia and physician-assisted suicide, and is used interchangeably with assisted dying. While some countries have legalized PAD, many others continue to deliberate, some remain silent, and many don’t engage on this issue. Physicians’ perspectives are central to these discussions, yet cross-national data remain limited. This study seeks to explore these perspectives and identify the factors that influence them.

Methods

We conducted an anonymous, online cross-sectional survey of physicians’ opinions across 17 countries (n = 107). The questionnaire assessed demographics, clinical background, experiences with PAD and ethical/legal attitudes using Likert scales, checkboxes and free-text responses. This was disseminated through professional networks using online platforms.

Results

Of the 107 respondents, 55 (51.4%) practiced in Asia, 42 (39.3%) in the United Kingdom, and 10 (9.3%) in the United States, Europe, Oceania or Africa. Over half (n = 55, 51.9%) were aged between 25-34 years and nearly a quarter (n = 25, 23.4%) had more than 20 years of clinical experience across diverse specialties. Majority (n = 58, 54.2%) supported legalization of PAD for mentally competent, terminally ill adults, with declining support for non-terminal physical suffering (n = 47, 43.9%) and psychological suffering (n = 27, 25.2%). While views diverged on whether PAD undermines doctor-patient trust, most agreed it could coexist with palliative care. Legislative preferences varied widely, with leading support for legalizing PAD with strict safeguards (n = 39, 36.4%), while 15 (14.0%) preferred that it remain illegal.

Discussion

The findings of our survey align with published single-country studies which showed that majority supported PAD in restricted contexts. Ethical, cultural and professional concerns persist, with tension between autonomy and non-maleficence being a central theme. There seems to be a notable cross-sectional diversity with strong opposition from Northeast Asia and conservative regions and more openness in the West. These results should be interpreted considering study limitations, including the relatively small sample size and uneven representation across countries.

Conclusion

Doctors across multiple countries demonstrate nuanced but generally supportive views of PAD under strict safeguards.

## Introduction

Death is an inevitable outcome of life, and a dignified death is desired by many. Physician-assisted death (PAD) has become a global debate about its ethical and legal justification. For the purpose of this paper, the term PAD is used interchangeably with assisted dying and encompasses both euthanasia and physician-assisted suicide. The distinction lies in the administration of the life-ending intervention. In euthanasia, it is carried out by the physician, whereas in physician-assisted suicide, the patient self-administers [1]. To date, only a few countries have legalized some form of PAD [2,3]. Many others continue to contest the issue within political, ethical, and medical realms, whilst the remaining stay silent on the matter. Most studies have examined opinions within a single country and often included other healthcare professionals or the public in their surveys [4,5]. This study aims to explore physicians’ perspectives on physician-assisted death across different countries and to identify demographic, cultural, and professional factors that influence these views.

## Materials and methods

All references to previously published studies were used solely to provide context and comparison for the findings of the present research. No copyrighted material, survey instruments, figures, or tables were reproduced or adapted from these sources. All references were accessed through publicly available or institutionally licensed sources. Ethical approval was deemed exempt as the study involved anonymous survey data with no identifiable personal information.

We conducted a cross-sectional survey using an online questionnaire developed in Google Forms (Google LLC, Mountain View, CA, USA). All survey items were developed de novo by the investigators to address the study’s objectives. The questionnaire was available only in English. We distributed it to doctors within our professional networks using online platforms, requesting that they share it further with colleagues to maximize our reach. Participation was voluntary and the survey was kept open for 14 days from 10th September 2025 to 23rd September 2025. 

There were 20 questions in total, 17 objective questions and three free-text questions. We assessed the demographics, clinical background, experiences in PAD and ethical and legal attitudes towards PAD. Collecting demographic information allowed comparison of cultural and geographical influences on attitudes towards PAD. Variables included age, country of practice and country of primary medical qualification. Clinical background questions include specialty, years and primary setting of practice. Experiences in PAD were assessed using questions with binary answers (“yes” or “no”) and the ethical and legal attitudes using a combination of multiple-choice questions, checkboxes and Likert scales. The Likert items assessed levels of agreement with statements, where 1 indicated 'strongly disagree', 3 indicated 'neutral', and 5 indicated 'strongly agree'. The final question was an optional free-text item inviting respondents to explain the reasons behind their opinions on PAD. The full survey questionnaire is provided in the Appendix.

Quantitative data were analyzed using Google Sheets. Descriptive statistics, including frequencies, percentages, and visual summaries, were generated to present the distribution of responses. This approach aligns with established guidance for descriptive analyses in cross-sectional survey research [6]. Qualitative free-text response was reviewed by two authors independently and thematically analyzed using an inductive approach, identifying recurring themes and patterns within the data [7]. All participants holding a primary medical qualification were included in the final analysis. One incomplete response was excluded and therefore not reflected in the results. Some survey item responses were omitted from the final analysis as they did not demonstrate clear patterns or add interpretive value to the study findings.

## Results

We received 107 responses from a total of 17 countries of practice and 26 countries of primary medical qualification. The demographics of the participants are shown in Table 1. Over half of the respondents (n = 55, 51.4%) practiced in Asia, 42 (39.3%) in the United Kingdom, and 10 (9.3%) in the United States, Europe, Oceania or Africa. 

**Table 1:** Demographic characteristics of participants (n = 107)

Variable	Category	n(%)
Age group	<25	2(1.9%)
	25-34	55 (51.4%)
	35-44	18 (16.8%)
	45-54	11(10.3%)
	≥55	21(19.6%)
Country of practice	Asia	55(51.4%)
	United Kingdom	42 (39.3%)
	Other (Europe, United States of America, Oceania, or Africa)	10(9.3%)
Country of primary medical qualification	Asia	85 (79.5%)
	United Kingdom	6 (5.6%)
	Other (Europe, United States of America, Oceania, or Africa)	16 (14.9%)

The largest professional group comprised those in medical specialties (n = 49, 45.8%), including five in oncology and two in palliative care. Fourteen (13.1%) respondents worked in emergency medicine, 10 (9.3%) in surgical specialties and 10 (9.3%) in general practice. Smaller groups included obstetrics and gynecology, psychiatry, intensive care, pediatrics, radiology, pathology, and other rotational specialties (n = 24, 22%). Percentages may not total 100% due to rounding. Over half (n = 55, 51.4%) were aged between 25-34 years old and nearly a quarter (n = 25, 23.4%) had more than 20 years of clinical experience. 

As shown in Table 2, most physicians (n = 58, 54.2%) supported the legalization of PAD in competent, terminally ill adults. While similar opinions were observed in the case of non-terminal intolerable physical suffering, views were starkly opposed in non-terminal intolerable psychological suffering. Conversely, a total of 41 (38.3%) objected to participating in PAD on moral or religious grounds, 31 of whom qualified in the eastern hemisphere. Many stated that physicians are meant to heal and preserve life, not end it. Some stated that physicians are not God to decide when death occurs, and others raised concerns about coercion, misuse, and inadequate funding for palliative services. 

**Table 2:** Distribution of Responses to Key Ethical Statements on Physician-Assisted Death (PAD) Likert scale - 1: strongly disagree, 3: neutral, 5: strongly agree

Question	1-2	3	4-5
PAD can be ethically justified in some circumstances.	27 (25.2%)	36 (33.6%)	44 (41.2%)
PAD should be legal for competent, terminally ill adults	23 (21.5%)	26 (24.3%)	58 (54.2%)
PAD should be legal for intolerable suffering due to physical ailments without a terminal prognosis	32 (29.9%)	28 (26.2%)	47 (43.9%)
PAD should be legal for intolerable suffering due to psychological reasons without a terminal prognosis.	51 (47.7%)	29 (27.1%)	27 (25.2%)
PAD undermines trust in the doctor–patient relationship.	34 (31.8%)	46 (42.9%)	27 (25.3%)
Palliative care and PAD can ethically coexist.	26 (24.3%)	20 (18.7%)	61 (57%)
Patient autonomy should take precedence over non-maleficence in end-of-life decisions.	20 (18.7%)	35 (32.7%)	52 (48.6%)
I would personally object to participating in PAD for moral/religious reasons.	38 (35.5%)	28 (26.2%)	41 (38.3%)

The optional free-text responses were categorized into seven overarching themes as evidenced in Table 3. Autonomy emerged as the most frequently cited ethical consideration (n = 23, 21.5%), with participants emphasizing the importance of patient choice and decision-making capacity. Governance-related concerns, including legal clarity, safeguards, and the potential for misuse, were identified in 12 responses (11.2%). A substantial proportion of participants did not provide a relevant free-text response (n = 39, 36.4%).

**Table 3:** Distribution of Themes in Qualitative Responses

Theme	N (%)
Autonomy	23 (21.5 %)
Quality of life	12 (11.2%)
Governance	12 (11.2%)
Non-maleficence	8 (7.5%)
Religious	12 (11.2%)
Patient factor	1 (0.9%)
No response	39 (36.5%)

**Figure 1:**
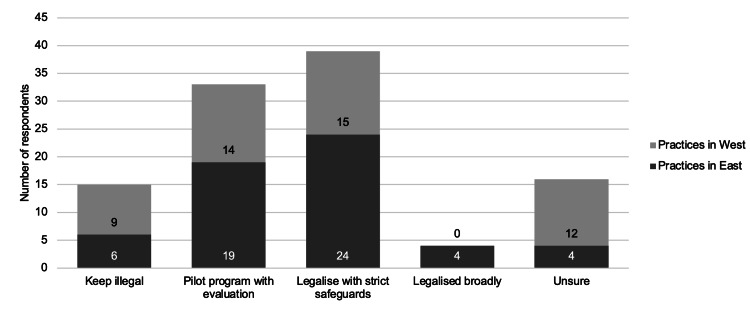
Summary of Legislation Preference

Twenty-three (21.5%) respondents disagreed with the statement “PAD should be legal for competent, terminally ill adults”. Of the 23, 14 (61%) felt it should remain illegal, six (26%) were unsure, two (9%) voted for legalizing with strict safeguards and one (4%) suggested piloting the program with evaluation. Notably, one respondent who initially agreed with the statement ultimately voted to keep PAD illegal, citing concerns about potential misuse. Figure 1 shows the legislation preferences of PAD with majority voting for it to be legalized with strict safeguards.

## Discussion

The concept of euthanasia can be traced back to ancient Greece, where the term originally referred to a “good death” rather than physician intervention [8]. The ethical and legal debate gained momentum in the late 20th century, when terms such as physician-assisted suicide and physician-assisted death entered mainstream bioethics [1,9]. The first formal legalization of euthanasia occurred in the Netherlands in 2001, marking a turning point in global discussions surrounding assisted dying [9].

Several recent studies reported similar results to ours, showing strong support for PAD in terminally ill patients, and decreasing support for non-terminal somatic illness and psychiatric disorders [10,11]. Individuals with psychological suffering are widely regarded as a vulnerable group, given the potential influence of their illness on autonomy, fluctuating capacity, and the risk of coercion [12]. This brings us back to the “slippery slope” argument that was raised during the 2005 House of Lords debate on the Assisted Dying for the Terminally Ill Bill, highlighting concerns of the eligibility criteria expanding and the potential abuse of vulnerable individuals [13].

A systematic review conducted by McCormack et al. (2012) on the attitudes of UK doctors towards PAD revealed markedly opposing perspectives influenced primarily by religious factors [4]. The evolution of these perceptions can be observed in the 2020 British Medical Association survey where 50% of UK doctors supported the legalization of prescribing life-ending medications. However, a significant number of respondents (45%) were unwilling to participate in the process. They cited conflicts with conscience, ethical beliefs and potential implications on the doctor-patient relationship [14]. These reasons closely align with the qualitative results of our study, with recurring tension between autonomy and non-maleficence, coupled with religious and moral considerations. Traditionally, the original Hippocratic Oath explicitly forbids giving a deadly drug in the context of “do no harm” [15]. This view has now shifted with bioethicists arguing that prolonging suffering can itself constitute harm [16]. The ongoing dilemma lies in how such ethical principles are translated into clinical practice, regulation, and legislation, particularly in balancing patient autonomy with adequate safeguards [12].

Religion and culture play a considerable role in physicians’ attitudes toward PAD. Multiple studies have consistently shown that religiosity strongly correlated with opposition to assisted dying, both in the UK and internationally [4,17,18]. Most major religions emphasize the sanctity of life and the moral prohibition against intentionally hastening death, whereas increasing secularization in Western societies is accompanied by growing acceptance of assisted dying [17,18]. Similarly, our survey showed that 41 (38.3%) respondents objected to participating in PAD due to moral or religious reasons, with the strongest disapproval of PAD observed in Northeast Asia and conservative regions of the world. While we assessed the country of practice and primary medical qualification, an additional question on religion and degree of religiosity could have provided deeper insight into the influences. 

Limitations

This study has several limitations. Participation was entirely voluntary, which may have introduced self-selection bias, as individuals with stronger opinions about physician-assisted death were perhaps more likely to respond. The overall sample size was relatively small, limiting the statistical power and generalizability of the findings. Additionally, there was uneven representation across countries and regions, with most responses originating from Asia and the United Kingdom, potentially influencing the observed trends. The questionnaire was available only in English which potentially excludes non-English-speaking physicians and introduces a selection bias. Future studies with larger and more geographically balanced samples would help to validate and expand upon these findings.

## Conclusions

This study highlights that doctors across multiple countries hold nuanced but generally supportive views toward PAD, particularly when implemented under strict safeguards and limited to competent, terminally ill adults. Cultural, religious, and ethical factors significantly influence these attitudes, with autonomy emerging as a central consideration in end-of-life decision-making. However, concerns remain regarding potential misuse, coercion, and the erosion of trust in the doctor-patient relationship. As discussions on legalization continue globally, it is essential that physicians’ perspectives inform policymaking to ensure that any future frameworks balance patient autonomy, professional integrity, and robust safeguards to protect both patients and practitioners.

## Appendices

**Table 4:** Survey Questionnaire

Question	Options of answers
Are you currently a licensed physician?	“Yes” or “No”
Country of practice	Dropdown - All countries included (Only allowed to select one)
University of undergraduate training	Free text
What specialty do you work in?	Free text
What is your age group?	Multiple choice question – 6 age categories and 1 “prefer not to say” option
Years of practice	Multiple choice question – 5 categories
Have you ever worked in a palliative care setting?	“Yes” or “No”
Practice setting	Checkboxes – 4 options and 1 “Other” with free text option
To your knowledge, is PAD currently legal in your country/region?	“Yes”, “No” or “Unsure”
Have you ever cared for patients who died under PAD or similar laws (eg. MAiD, VAD, euthanasia)?	“Yes”, “No” or “Prefer not to say”
PAD can be ethically justified in some circumstances	Likert scale – 1= strongly disagree, 3 = neutral, 5 = strongly agree
PAD should be legal for competent, terminally ill adults	Likert scale – 1= strongly disagree, 3 = neutral, 5 = strongly agree
PAD should be legal for intolerable suffering due to physical ailments without a terminal prognosis.	Likert scale – 1= strongly disagree, 3 = neutral, 5 = strongly agree
PAD should be legal for intolerable psychological suffering due to psychological reasons without a terminal prognosis	Likert scale – 1= strongly disagree, 3 = neutral, 5 = strongly agree
PAD undermines trust in the doctor-patient relationship	Likert scale – 1= strongly disagree, 3 = neutral, 5 = strongly agree
PAD and palliative care can ethically coexist	Likert scale – 1= strongly disagree, 3 = neutral, 5 = strongly agree
Patient autonomy should take precedence over non-maleficence in end-of-life decisions	Likert scale – 1= strongly disagree, 3 = neutral, 5 = strongly agree
I would personally object to participating in PAD for moral/religious reasons	Likert scale – 1= strongly disagree, 3 = neutral, 5 = strongly agree
If PAD were legal where you practice, which roles would you be willing to take?	Checkboxes -Discuss PAD neutrally upon patient inquiry/Provide information/referral to PAD pathways /Complete eligibility assessments /Prescribe life-ending medication /Be present at ingestion/administration /None of the above
Which of the following do you think is essential when considering PAD?	Checkboxes -Second independent physician assessment /Mandatory palliative-care consult/Mandatory psychiatric assessment when indicated/ Waiting (cool-off) period/ Capacity assessment & informed consent documentation/ Age ≥ 18/ Terminal illness prognosis (≤6–12 months)/ Refractory suffering despite optimal care (not necessarily terminal)/ All of the above/ Other
If your country changed PAD law today, your preference would be...	Multiple choice question - Keep illegal/ Pilot program with evaluation /Legalize with strict safeguards/ Legalize broadly Unsure
In your own words, what ethical considerations most influence your view on PAD?	Long answer text (not a mandatory question)
